# Supplementary material for: Influence of Climatic Region and Feedstuff Type on the Co-Occurrence and Contamination Profiles of 54 Mycotoxins in European Grains and Forages: A Seven-Year Survey
Source: Toxins (Basel). 2025 Dec 20;18(1):5. doi: 10.3390/toxins18010005 (PMC12845635; doi:10.3390/toxins18010005)
Supplement: Supplementary file 1 [file toxins-18-00005-s001.zip › toxins-4034306-supplementary.pdf]

toxins

Article

Supplementary Material: Influence of Climatic Region and Feedstuff Type on the Co-Occurrence and Contamination Profiles of 54 Mycotoxins in European Grains and Forages: A Seven-Year Survey

Supplementary Table S1. Concentration of mycotoxins (µg/kg) by group in barley measured by ultra pressure liquid chromatography tandem mass spectrometry from different European climatic regions over seven harvest years. Median, mean and quartiles were calculated from concentrations above the limit of quantification (LOQ).

Group[1]	Climatic Region	Mean	SD[2]	Q1[2]	Median	Q3[2]	Maximum	*P* value[3]
AFs	Continental	0.03	0.21	0.00	0.00	0.00	2.17	0.681
	Mediterranean	0.04	0.27	0.00	0.00	0.00	1.82	
	Nordic	0.00	0.00	0.00	0.00	0.00	0.00	
	Oceanic	0.01	0.16	0.00	0.00	0.00	2.01	
OTs/CIT	Continental	0.27	3.83	0.00	0.00	0.00	60.70	0.911
	Mediterranean	0.87	5.86	0.00	0.00	0.00	39.31	
	Nordic	0.00	0.00	0.00	0.00	0.00	0.00	
	Oceanic	1.52	29.48	0.00	0.00	0.00	584.34	
B Tricho.	Continental	341.41	1,179.63	0.00	45.69	162.85	14,074.48	<0.005
	Mediterranean	303.40	983.02	0.00	70.13	201.19	6,459.48	
	Nordic	3,783.15	6,997.05	480.84	1,476.99	3,428.30	28,987.66	
	Oceanic	119.94	195.30	0.00	49.64	154.16	1,853.14	
A Tricho.	Continental	26.22	66.38	0.00	0.00	21.11	516.51	0.048
	Mediterranean	9.33	23.26	0.00	0.00	10.90	143.32	
	Nordic	49.70	61.45	0.96	29.24	81.13	240.77	
	Oceanic	21.78	49.53	0.00	0.00	23.54	450.60	
FUMs	Continental	6.59	26.98	0.00	0.00	0.00	380.41	0.516
	Mediterranean	11.98	24.21	0.00	0.00	20.97	150.22	
	Nordic	7.37	14.65	0.00	0.00	7.49	49.21	
	Oceanic	6.84	19.35	0.00	0.00	0.00	155.19	
ZEA	Continental	4.52	31.71	0.00	0.00	0.00	424.46	<0.005
	Mediterranean	0.04	0.29	0.00	0.00	0.00	1.93	
	Nordic	166.09	260.42	0.00	51.94	207.65	924.65	
	Oceanic	0.59	4.85	0.00	0.00	0.00	60.23	
FA	Continental	4.90	39.63	0.00	0.00	0.00	547.74	0.207
	Mediterranean	5.78	35.76	0.00	0.00	0.00	239.70	
	Nordic	0.30	1.26	0.00	0.00	0.00	5.34	
	Oceanic	0.88	6.91	0.00	0.00	0.00	99.86	
Emerg.	Continental	163.95	458.51	1.02	17.88	90.32	3,247.41	<0.005
	Mediterranean	59.91	173.60	3.22	12.43	29.49	1,036.00	
	Nordic	1,560.89	1,439.75	692.51	1,279.78	1,754.40	5,144.72	
	Oceanic	374.81	679.76	25.57	107.16	410.81	4,611.46	
Pen.	Continental	1.80	16.38	0.00	0.00	0.00	191.72	0.087
	Mediterranean	0.00	0.00	0.00	0.00	0.00	0.00	
	Nordic	1.85	6.60	0.00	0.00	0.00	27.75	
	Oceanic	8.49	46.54	0.00	0.00	0.00	548.67	
Asp.	Continental	1.14	5.86	0.00	0.00	0.00	58.31	0.221
	Mediterranean	1.02	5.92	0.00	0.00	0.00	39.31	

	Nordic	2.80	9.75	0.00	0.00	0.00	41.42	
	Oceanic	0.64	3.67	0.00	0.00	0.00	44.63	
Ergot	Continental	12.82	174.43	0.00	0.00	0.00	2,768.10	0.315
	Mediterranean	2.66	17.87	0.00	0.00	0.00	119.85	
	Nordic	90.93	333.22	0.00	0.00	2.74	1,418.38	
	Oceanic	41.32	300.01	0.00	0.00	0.00	3,055.04	

[1]AFs: total aflatoxins; OTs/CIT: ochratoxins/citrinin; B Tricho.: B trichothecenes; A Tricho.: A trichothecenes; FUMs: total fumonisins; ZEA: zearalenone; FA: fusaric acid; Emerg.: emerging mycotoxins; Pen.: *Penicillium* mycotoxins; Asp.: *Aspergillus* mycotoxins; Ergot: ergot alkaloids. [2]SD: standard deviation; Q1: first quartile; Q3: third quartile. [3] *P*-value for the mean for climatic region effect within each group.

Supplementary Table S2. Concentration of mycotoxins (μg/kg) by group in maize measured by ultra pressure liquid chromatography tandem mass spectrometry from different European climatic regions over seven harvest years. Median, mean and quartiles were calculated from concentrations above the limit of quantification (LOQ).

Group[1]	Climatic Region	Mean	SD[2]	Q1[2]	Median	Q3[2]	Maximum	*P* value[3]
AFs	Continental	9.83	42.66	0.00	0.00	0.00	265.51	0.245
	Mediterranean	3.58	31.07	0.00	0.00	0.00	466.10	
	Nordic							
	Oceanic	0.00	0.00	0.00	0.00	0.00	0.00	
OTs/CIT	Continental	3.36	19.15	0.00	0.00	0.00	167.80	0.105
	Mediterranean	0.58	7.31	0.00	0.00	0.00	118.03	
	Nordic							
	Oceanic	0.00	0.00	0.00	0.00	0.00	0.00	
B Tricho.	Continental	89.08	187.81	0.00	17.04	71.20	1,023.93	<0.005
	Mediterranean	166.34	471.74	0.00	29.04	98.06	3,974.41	
	Nordic							
	Oceanic	868.07	777.73	264.66	798.56	1,181.07	2,832.32	
A Tricho.	Continental	17.54	58.93	0.00	0.00	1.71	458.64	<0.005
	Mediterranean	1.26	7.64	0.00	0.00	0.00	73.49	
	Nordic							
	Oceanic	33.51	100.92	0.00	0.00	12.54	414.51	
FUMs	Continental	2,265.06	5,228.93	12.72	347.46	1,273.25	29,424.52	0.0431
	Mediterranean	1,928.21	3,005.56	177.21	862.67	2,094.47	20,392.29	
	Nordic							
	Oceanic	182.07	347.25	0.00	31.10	173.27	1,553.55	
ZEA	Continental	5.16	30.45	0.00	0.00	0.00	258.62	0.0894
	Mediterranean	10.30	86.47	0.00	0.00	0.00	1,198.24	
	Nordic							
	Oceanic	43.83	43.07	0.00	36.51	58.67	150.88	
FA	Continental	161.05	191.25	38.58	95.10	212.34	901.66	0.486
	Mediterranean	204.79	320.47	35.21	84.45	186.62	2,050.33	
	Nordic							
	Oceanic	178.52	368.09	34.35	61.81	146.93	1,648.34	
Emerg.	Continental	283.91	697.50	15.00	64.08	174.18	4,750.76	0.316
	Mediterranean	346.99	835.37	20.27	97.77	314.90	8,844.40	
	Nordic							
	Oceanic	100.95	154.61	5.37	35.60	134.34	507.68	
Pen.	Continental	2.43	13.46	0.00	0.00	0.00	98.61	0.824
	Mediterranean	4.69	33.42	0.00	0.00	0.00	484.25	
	Nordic							
	Oceanic	4.57	15.48	0.00	0.00	0.00	72.68	
Asp.	Continental	17.18	91.76	0.00	0.00	0.00	795.51	0.05
	Mediterranean	2.73	27.43	0.00	0.00	0.00	451.85	
	Nordic							
	Oceanic	0.00	0.00	0.00	0.00	0.00	0.00	
Ergot	Continental	0.00	0.00	0.00	0.00	0.00	0.00	0.561

	Mediterranean	0.02	0.23	0.00	0.00	0.00	2.39	
	Nordic							
	Oceanic	0.00	0.00	0.00	0.00	0.00	0.00	

[1]AFs: total aflatoxins; OCH/CIT: ochratoxins/citrinin; B Tricho.: type B trichothecenes; A Tricho.: type A trichothecenes; FUMs: total fumonisins; ZEA: zearalenone; FA: fusaric acid; Emerg.: emerging mycotoxins; Pen.: *Penicillium* mycotoxins; Asp.: *Aspergillus* mycotoxins; Ergot: ergot alkaloids. [2] SD: standard deviation; Q1: first quartile; Q3: third quartile. [3] *P*-value for the mean for climatic region effect within each group.

Supplementary Table S3. Concentration of mycotoxins (μg/kg) by group in wheat measured by ultra pressure liquid chromatography tandem mass spectrometry from different European climatic regions over seven harvest years. Median, mean and quartiles were calculated from concentrations above the limit of quantification (LOQ).

Group[1]	Climatic Region	Mean	SD[2]	Q1[2]	Median	Q3[2]	Maximum	*P* value[3]
AFs	Continental	0.01	0.11	0.00	0.00	0.00	1.20	0.789
	Mediterranean	0.00	0.00	0.00	0.00	0.00	0.00	
	Nordic							
	Oceanic	0.01	0.16	0.00	0.00	0.00	2.98	
OTs/CIT	Continental	1.06	15.80	0.00	0.00	0.00	278.60	0.559
	Mediterranean	0.00	0.00	0.00	0.00	0.00	0.00	
	Nordic							
	Oceanic	0.27	3.11	0.00	0.00	0.00	49.11	
B Tricho.	Continental	262.14	733.94	0.00	33.50	149.49	7,281.83	<0.005
	Mediterranean	228.61	504.72	0.00	24.52	170.41	2,506.77	
	Nordic							
	Oceanic	110.66	259.48	0.00	29.66	92.28	2,382.64	
A Tricho.	Continental	4.16	13.06	0.00	0.00	0.00	132.21	0.266
	Mediterranean	1.85	5.95	0.00	0.00	0.00	41.24	
	Nordic							
	Oceanic	3.28	10.66	0.00	0.00	0.00	92.54	
FUMs	Continental	6.48	17.46	0.00	0.00	0.00	198.57	0.037
	Mediterranean	6.42	10.39	0.00	0.00	13.13	42.83	
	Nordic							
	Oceanic	12.02	38.87	0.00	0.00	11.99	414.06	
ZEA	Continental	2.51	20.06	0.00	0.00	0.00	307.83	0.742
	Mediterranean	1.09	4.24	0.00	0.00	0.00	21.17	
	Nordic							
	Oceanic	1.84	12.16	0.00	0.00	0.00	171.52	
FA	Continental	1.15	5.15	0.00	0.00	0.00	41.68	0.807
	Mediterranean	0.84	5.11	0.00	0.00	0.00	39.80	
	Nordic							
	Oceanic	0.76	9.79	0.00	0.00	0.00	165.59	
Emerg.	Continental	27.66	70.11	1.22	7.08	19.85	876.73	<0.005
	Mediterranean	14.94	44.12	0.00	3.24	14.01	340.54	
	Nordic							
	Oceanic	67.41	231.29	5.27	14.95	45.18	3,114.48	
Pen.	Continental	3.37	40.65	0.00	0.00	0.00	517.41	0.732
	Mediterranean	0.57	4.69	0.00	0.00	0.00	38.99	
	Nordic							
	Oceanic	2.09	19.53	0.00	0.00	0.00	270.08	
Asp.	Continental	0.37	3.46	0.00	0.00	0.00	57.74	0.145
	Mediterranean	0.14	0.79	0.00	0.00	0.00	4.72	
	Nordic							
	Oceanic	0.02	0.30	0.00	0.00	0.00	4.39	
Ergot	Continental	1.37	10.06	0.00	0.00	0.00	149.11	0.064
	Mediterranean	0.00	0.00	0.00	0.00	0.00	0.00	
	Nordic							
	Oceanic	15.71	122.46	0.00	0.00	0.00	1,753.23	

[1]AFs: total aflatoxins; OCH/CIT: ochratoxins/citrinin; B Tricho.: type B trichothecenes; A Tricho.: type A trichothecenes; FUMs: total fumonisins; ZEA: zearalenone; FA: fusaric acid; Emerg.: emerging mycotoxins; Pen.: *Penicillium* mycotoxins; Asp.: *Aspergillus* mycotoxins; Ergot: ergot alkaloids. [2] SD: standard deviation; Q1: first quartile; Q3: third quartile. [3] *P*-value for the mean for climatic region effect within each group.

Supplementary Table S4. Concentration of mycotoxins (μg/kg) by group in maize silage measured by ultra pressure liquid chromatography tandem mass spectrometry by European climatic regions over seven harvest years. Median, mean and quartiles were calculated from concentrations above the limit of quantification (LOQ).

Group[1]	Climatic Region	Mean	SD[2]	Q1[2]	Median	Q3[2]	Maximum	*P* value[3]
AFs	Continental	0.04	0.37	0.00	0.00	0.00	3.57	0.006
	Mediterranean	7.64	30.77	0.00	0.00	0.00	161.94	
	Nordic							
	Oceanic	1.47	14.94	0.00	0.00	0.00	152.37	
OTs/CIT	Continental	2.24	29.34	0.00	0.00	0.00	383.63	0.627
	Mediterranean	3.46	25.19	0.00	0.00	0.00	183.40	
	Nordic							
	Oceanic	0.00	0.00	0.00	0.00	0.00	0.00	
B Tricho.	Continental	2,169.62	7,351.81	329.82	799.18	1,808.24	79,024.12	0.089
	Mediterranean	397.82	704.50	106.99	204.28	446.43	4,903.30	
	Nordic							
	Oceanic	1,281.09	1,672.37	331.00	643.82	1,705.34	11,374.18	
A Tricho.	Continental	102.66	346.51	0.00	0.00	97.37	4,265.08	0.022
	Mediterranean	44.77	203.01	0.00	0.00	0.00	1,222.34	
	Nordic							
	Oceanic	13.18	40.69	0.00	0.00	0.00	242.66	
FUMs	Continental	258.87	569.18	0.00	0.00	251.41	4,779.18	<0.005
	Mediterranean	788.87	1,273.35	104.53	392.40	952.01	8,122.59	
	Nordic							
	Oceanic	33.38	118.40	0.00	0.00	0.00	754.81	
ZEA	Continental	80.49	263.01	0.00	0.00	0.00	1,911.70	0.169
	Mediterranean	13.83	78.83	0.00	0.00	0.00	546.14	
	Nordic							
	Oceanic	67.23	204.92	0.00	0.00	0.00	1,420.64	
FA	Continental	684.22	699.92	182.41	499.79	981.14	4,491.00	0.011
	Mediterranean	1,346.80	4,857.45	376.16	642.87	1,052.26	35,870.67	
	Nordic							
	Oceanic	313.99	371.34	66.09	161.14	451.99	1,802.18	
Emerg.	Continental	53.38	78.64	4.69	25.48	71.18	538.86	0.078
	Mediterranean	39.66	87.57	0.00	3.11	29.04	495.44	
	Nordic							
	Oceanic	77.15	149.64	1.04	30.29	80.51	1,197.93	
Pen.	Continental	26.51	104.50	0.00	0.00	2.83	993.14	0.948
	Mediterranean	24.32	65.35	0.00	0.00	0.00	305.54	
	Nordic							
	Oceanic	29.41	100.92	0.00	0.00	0.00	863.96	
Asp.	Continental	1.44	12.38	0.00	0.00	0.00	145.13	0.423
	Mediterranean	2.61	10.75	0.00	0.00	0.00	60.42	
	Nordic							
	Oceanic	8.79	79.19	0.00	0.00	0.00	805.30	
Ergot	Continental	2.35	30.69	0.00	0.00	0.00	401.34	0.676
	Mediterranean	0.20	1.43	0.00	0.00	0.00	10.39	
	Nordic							
	Oceanic	0.16	1.41	0.00	0.00	0.00	14.16	

[1]AFs: total aflatoxins; OCH/CIT: ochratoxins/citrinin; B Tricho.: type B trichothecenes; A Tricho.: type A trichothecenes; FUMs: total fumonisins; ZEA: zearalenone; FA: fusaric acid; Emerg.: emerging mycotoxins; Pen.: *Penicillium* mycotoxins; Asp.: *Aspergillus* mycotoxins; Ergot: ergot alkaloids. [2] SD: standard deviation; ; Q1: first quartile; Q3: third quartile. [3] *P*-value for the mean for climatic region effect within each mycotoxin.

Supplementary Table S5. Concentration of mycotoxins (μg/kg) by group in grass silage measured by ultra pressure liquid chromatography tandem mass spectrometry from different European climatic regions over seven harvest years. Median, mean and quartiles were calculated from concentrations above the limit of quantification (LOQ).

Group[1]	Climatic Region	Mean	SD[2]	Q1[2]	Median	Q3[2]	Maximum	*P* value[3]
AFs	Continental	6.20	35.32	0.00	0.00	0.00	293.30	0.112
	Mediterranean	0.00		0.00	0.00	0.00	0.00	
	Nordic	0.00	0.00	0.00	0.00	0.00	0.00	
	Oceanic	0.74	12.44	0.00	0.00	0.00	210.04	
OTs/CIT	Continental	0.00	0.00	0.00	0.00	0.00	0.00	0.888
	Mediterranean	0.00		0.00	0.00	0.00	0.00	
	Nordic	0.00	0.00	0.00	0.00	0.00	0.00	
	Oceanic	0.59	10.04	0.00	0.00	0.00	169.42	
B Tricho.	Continental	108.88	320.58	0.00	7.46	71.48	2,620.84	0.857
	Mediterranean	0.00		0.00	0.00	0.00	0.00	
	Nordic	84.23	69.60	34.68	60.29	129.06	217.97	
	Oceanic	127.70	300.49	0.00	35.01	91.40	2,973.27	
A Tricho.	Continental	2.18	11.27	0.00	0.00	0.00	105.30	0.376
	Mediterranean	22.35		22.35	22.35	22.35	22.35	
	Nordic	0.00	0.00	0.00	0.00	0.00	0.00	
	Oceanic	1.49	14.10	0.00	0.00	0.00	207.90	
FUMs	Continental	12.44	61.93	0.00	0.00	0.00	602.70	0.585
	Mediterranean	0.00		0.00	0.00	0.00	0.00	
	Nordic	0.00	0.00	0.00	0.00	0.00	0.00	
	Oceanic	7.63	30.91	0.00	0.00	0.00	274.65	
ZEA	Continental	5.02	43.79	0.00	0.00	0.00	491.51	0.960
	Mediterranean	0.00		0.00	0.00	0.00	0.00	
	Nordic	0.00	7.71	0.00	0.00	0.00	0.00	
	Oceanic	3.93	33.78	0.00	0.00	0.00	409.33	
FA	Continental	30.98	41.40	0.00	17.00	40.30	221.70	0.119
	Mediterranean	92.58		92.58	92.58	92.58	92.58	
	Nordic	65.02	57.43	31.58	43.30	88.06	175.90	
	Oceanic	43.48	83.05	0.00	11.25	41.85	546.14	
Emerg.	Continental	33.31	62.29	0.00	10.59	38.12	526.16	<0.005
	Mediterranean	129.11		129.11	129.11	129.11	129.11	
	Nordic	28.69	40.04	9.27	16.85	30.84	164.85	
	Oceanic	10.55	27.96	0.00	0.00	9.86	214.45	
Pen.	Continental	156.73	511.78	0.00	0.00	77.98	4679.75	0.001
	Mediterranean	0.00		0.00	0.00	0.00	0.00	
	Nordic	208.82	330.15	0.00	0.00	337.97	798.83	
	Oceanic	361.79	544.12	0.00	114.45	534.97	3,945.90	
Asp.	Continental	0.29	2.03	0.00	0.00	0.00	21.41	0.719
	Mediterranean	0.00		0.00	0.00	0.00	0.00	
	Nordic	0.99	2.86	0.00	0.00	0.00	10.48	
	Oceanic	0.21	2.81	0.00	0.00	0.00	46.60	
Ergot	Continental	6.69	76.81	0.00	0.00	0.00	982.61	0.798
	Mediterranean	10.04		10.04	10.04	10.04	10.04	
	Nordic	16.99	45.06	0.00	0.00	0.00	139.63	
	Oceanic	15.41	99.32	0.00	0.00	0.00	989.29	

[1]AFs: total aflatoxins; OTs/CIT: ochratoxins/citrinin; B Tricho.: B trichothecenes; A Tricho.: A trichothecenes; FUMs: total fumonisins; ZEA: zearalenone; FA: fusaric acid; Emerg.: emerging mycotoxins; Pen.: *Penicillium* mycotoxins; Asp.: *Aspergillus* mycotoxins; Ergot: ergot alkaloids. [2] SD: standard deviation; Q1: first quartile; Q3: third quartile. [3] *P*-value for the mean for climatic region effect within each group.

Supplementary Table S6. Mean concentration (μg/kg) of mycotoxin groups across seven years (2018 to 2024) in five European feedstuffs.

	Barley		Maize		Wheat		Maize Silage		Grass Silage	
Year	Mean	CI[2]	Mean	CI	Mean	CI	Mean	CI	Mean	CI
AFs[1]										
2018	0	0	0.31	0.41	0	0	38.26	39.22	0.00	0.00
2019	0	0	0.23	0.32	0.06	0.06	0	0	12.36	24.22
2020	0.09	0.09	0.19	0.28	0.05	0.08	2.05	2.84	41.03	37.40
2021	0.04	0.04	0.69	0.70	0	0	0.06	0.09	0.00	0.00
2022	0	0	12.26	13.62	0	0	0	0	1.24	1.58
2023	0	0	1.49	1.12	0	0	0	0	0.00	0.00
2024	0	0	15.64	13.92	0	0	0	0	0.00	0.00
P value	0.055		<0.005		0.006		0.006		<0.005	
OTs/CIT[1]										
2018	16.54	30.15	0.00	0.00	0.00	0.00	15.28	29.95	0.00	0.00
2019	0.24	0.47	0.00	0.00	0.24	0.33	0.00	0.00	0.00	0.00
2020	0.00	0.00	0.26	0.51	0.62	1.22	0.00	0.00	0.00	0.00
2021	0.05	0.09	1.80	3.23	0.43	0.63	0.00	0.00	0.00	0.00
2022	0.00	0.00	0.95	1.06	0.00	0.00	7.67	15.04	1.64	3.22
2023	0.42	0.83	0.55	1.08	0.00	0.00	0.00	0.00	0.00	0.00
2024	0.00	0.00	3.32	6.09	2.40	4.41	0.00	0.00	0.00	0.00
P value	0.835		0.193		0.003		0.584		0.742	
B Tricho.[1]										
2018	290.03	335.57	17.30	25.54	170.16	120.63	444.15	183.45	104.23	204.29
2019	702.08	527.11	15.49	18.47	766.71	427.30	343.08	133.56	124.47	187.97
2020	225.41	82.72	234.19	129.96	216.61	73.42	1,031.22	367.89	305.05	218.59
2021	118.38	54.49	198.49	84.96	154.17	76.80	999.01	353.14	112.35	49.58
2022	86.65	42.22	245.10	142.25	38.06	29.27	800.17	336.41	94.50	38.44
2023	631.59	455.96	180.07	93.26	95.96	42.09	3,050.06	1,842.59	56.87	32.19
2024	339.81	203.92	167.04	136.94	254.82	76.03	1,370.08	452.44	183.69	77.73
P value	0.447		0.093		0.016		<0.005		0.003	
A Tricho.[1]										
2018	0.41	0.79	2.33	4.57	1.89	2.12	224.89	226.57	9.73	16.61
2019	4.61	4.04	4.10	3.94	0.19	0.27	2.54	3.33	8.11	9.09
2020	3.54	3.23	0.96	1.15	1.20	1.17	0.00	0.00	0.42	0.83
2021	10.47	6.53	4.17	3.97	1.16	1.06	30.93	24.75	0.00	0.00
2022	32.79	13.42	4.44	6.58	1.23	0.90	24.57	16.02	0.05	0.07
2023	44.24	12.09	17.74	15.60	4.38	2.23	137.86	85.06	1.90	3.12
2024	31.04	7.70	5.50	4.93	10.74	2.83	33.94	15.69	2.75	2.46
P value	0.175		0.005		<0.005		<0.005		0.063	
FUMs[1]										
2018	0.00	0.00	1301.50	1412.47	0.44	0.61	501.62	345.52	0.00	0.00
2019	0.00	0.00	2555.26	2650.64	1.08	0.84	0.00	0.00	0.00	0.00
2020	31.07	7.06	975.71	284.13	22.15	5.48	461.08	339.62	15.77	6.32
2021	3.44	4.48	2406.26	709.45	1.66	0.69	453.99	184.78	2.77	5.42
2022	0.26	0.36	1942.37	901.46	0.00	0.00	218.09	118.32	0.00	0.00
2023	4.00	2.22	1880.13	777.06	11.80	7.77	129.16	70.46	15.03	12.79
2024	11.85	3.15	1609.13	876.95	20.60	3.16	38.68	21.23	15.17	5.67
P value	0.357		0.004		<0.005		<0.005		0.051	
ZEA[1]										
2018	0.00	0.00	0.00	0.00	0.46	0.90	0.00	0.00	0.00	0.00
2019	0.00	0.00	0.00	0.00	2.30	3.86	0.00	0.00	0.00	0.00

Year	Barley		Maize		Wheat		Maize Silage		Grass Silage	
	Mean	CI[2]	Mean	CI	Mean	CI	Mean	CI	Mean	CI
2020	2.43	2.20	11.11	7.79	3.40	2.90	16.50	24.04	0.00	0.00
2021	0.58	0.81	3.23	4.11	2.21	3.52	2.78	5.45	0.57	1.12
2022	0.58	0.67	31.08	33.20	0.13	0.25	119.26	86.90	4.77	9.35
2023	23.88	18.12	7.54	6.97	1.73	1.75	137.96	61.42	7.60	8.40
2024	4.51	4.21	5.73	5.73	3.44	2.91	19.43	30.91	3.69	4.90
P value	0.241		0.000		0.001		0.738		0.875	
FA[1]										
2018	6.31	12.36	38.44	16.31	0.00	0.00	266.12	195.76	132.10	79.91
2019	0.73	1.43	131.77	108.16	1.43	1.71	411.67	262.97	197.40	57.08
2020	0.57	0.78	177.95	50.94	2.56	4.16	1230.02	1423.01	96.74	24.15
2021	3.71	3.65	350.87	71.58	1.52	1.20	777.53	87.13	99.12	21.22
2022	4.93	9.50	172.99	74.92	0.08	0.16	504.70	129.54	25.09	5.16
2023	1.60	1.12	114.39	47.84	0.09	0.17	576.14	160.93	13.39	4.03
2024	0.76	0.57	97.25	38.91	1.12	0.76	325.14	143.83	4.50	4.75
P value	<0.005		0.439		0.743		0.187		<0.005	
Emerg.[1]										
2018	6.63	8.70	24.82	25.68	3.62	2.54	55.62	81.54	0.00	0.00
2019	162.94	143.01	186.14	232.30	12.62	10.09	4.60	6.36	20.25	22.83
2020	338.20	120.18	118.68	34.04	88.53	42.16	21.16	12.56	5.13	3.57
2021	146.73	47.15	420.02	144.56	23.33	7.15	17.80	11.14	0.75	1.12
2022	273.61	90.67	517.88	257.36	18.89	6.12	110.80	23.80	36.62	7.74
2023	686.84	184.73	179.01	117.34	43.30	12.24	98.02	29.91	31.51	11.49
2024	258.68	79.10	339.19	248.70	99.70	62.37	22.98	10.67	5.79	3.95
P value	0.015		<0.005		<0.005		<0.005		<0.005	
Pen.[1]										
2018	2.95	3.03	0.25	0.50	1.10	1.72	0.00	0.00	191.52	176.67
2019	2.23	4.38	0.00	0.00	0.00	0.00	0.00	0.00	315.27	247.77
2020	2.70	5.29	3.08	3.99	0.00	0.00	41.97	34.08	178.68	204.05
2021	1.56	1.68	6.75	9.48	0.15	0.21	29.28	14.42	386.22	165.18
2022	2.30	4.39	3.60	4.68	0.00	0.00	19.66	11.12	234.29	81.49
2023	15.94	10.32	5.66	4.73	8.71	8.64	32.93	27.26	322.30	105.49
2024	4.69	6.65	0.27	0.29	1.86	3.27	6.94	5.83	241.98	76.01
P value	0.882		0.601		0.014		0.107		0.422	
Asp.[1]										
2018	3.47	3.64	0.60	1.17	0.10	0.20	32.92	25.43	0.00	0.00
2019	2.83	3.67	0.00	0.00	0.00	0.00	0.79	1.55	0.06	0.11
2020	2.52	1.64	0.38	0.53	0.42	0.47	1.47	2.05	0.00	0.00
2021	1.12	0.76	0.55	0.34	0.17	0.14	10.44	19.98	0.75	1.31
2022	0.00	0.00	14.65	19.33	0.00	0.00	0.00	0.00	0.50	0.52
2023	0.10	0.20	1.37	1.49	0.37	0.64	0.00	0.00	0.03	0.07
2024	0.20	0.16	20.90	24.88	0.02	0.03	0.02	0.04	0.12	0.16

Year	Barley		Maize		Wheat		Maize Silage		Grass Silage	
	**Mean**	**CI[2]**	**Mean**	**CI**	**Mean**	**CI**	**Mean**	**CI**	**Mean**	**CI**
P value	0.164		0.241		<0.005		0.726		0.502	
Ergot[1]										
2018	80.40	157.58	0.00	0.00	0.11	0.21	0.00	0.00	21.02	32.02
2019	0.32	0.31	0.12	0.23	0.04	0.08	2.10	3.44	3.26	2.01
2020	30.07	56.20	0.00	0.00	1.56	1.88	0.21	0.42	0.00	0.00
2021	5.12	4.68	0.05	0.06	1.49	1.79	5.08	9.96	0.00	0.00
2022	54.90	59.73	0.00	0.00	11.50	11.31	0.00	0.00	3.38	3.67
2023	4.30	4.19	0.00	0.00	16.14	22.22	0.00	0.00	35.96	28.12
2024	61.15	63.67	0.00	0.00	13.20	15.43	0.00	0.00	3.60	5.09
P value	0.190		0.801		0.214		0.604		0.041	

[1]AFs: total aflatoxins; OTs/CIT: ochratoxins/citrinin; B Tricho.: B trichothecenes; A Tricho.: A trichothecenes; FUMs: total fumonisins; ZEA: zearalenone; FA: fusaric acid; Emerg.: emerging mycotoxins; Pen.: *Penicillium* mycotoxins; Asp.: *Aspergillus* mycotoxins; Ergot: ergot alkaloids. [2] CI: 95% confidence interval.
